# Reconstructing genome trees of prokaryotes using overlapping genes

**DOI:** 10.1186/1471-2105-11-102

**Published:** 2010-02-24

**Authors:** Chih-Hsien Cheng, Chung-Han Yang, Hsien-Tai Chiu, Chin Lung Lu

**Affiliations:** 1 Institute of Bioinformatics and Systems Biology, National Chiao Tung University, Hsinchu 300, Taiwan; 2 Department of Biological Science and Technology, National Chiao Tung University, Hsinchu 300, Taiwan

## Abstract

**Background:**

Overlapping genes (OGs) are defined as adjacent genes whose coding sequences overlap partially or entirely. In fact, they are ubiquitous in microbial genomes and more conserved between species than non-overlapping genes. Based on this property, we have previously implemented a web server, named OGtree, that allows the user to reconstruct genome trees of some prokaryotes according to their pairwise OG distances. By analogy to the analyses of gene content and gene order, the OG distance between two genomes we defined was based on a measure of combining OG content (i.e., the normalized number of shared orthologous OG pairs) and OG order (i.e., the normalized OG breakpoint distance) in their whole genomes. A shortcoming of using the concept of breakpoints to define the OG distance is its inability to analyze the OG distance of multi-chromosomal genomes. In addition, the amount of overlapping coding sequences between some distantly related prokaryotic genomes may be limited so that it is hard to find enough OGs to properly evaluate their pairwise OG distances.

**Results:**

In this study, we therefore define a new OG order distance that is based on more biologically accurate rearrangements (e.g., reversals, transpositions and translocations) rather than breakpoints and that is applicable to both uni-chromosomal and multi-chromosomal genomes. In addition, we expand the term "gene" to include both its coding sequence and regulatory regions so that two adjacent genes whose coding sequences or regulatory regions overlap with each other are considered as a pair of overlapping genes. This is because overlapping of regulatory regions of distinct genes suggests that the regulation of expression for these genes should be more or less interrelated. Based on these modifications, we have reimplemented our OGtree as a new web server, named OGtree2, and have also evaluated its accuracy of genome tree reconstruction on a testing dataset consisting of 21 Proteobacteria genomes. Our experimental results have finally shown that our current OGtree2 indeed outperforms its previous version OGtree, as well as another similar server, called BPhyOG, significantly in the quality of genome tree reconstruction, because the phylogenetic tree obtained by OGtree2 is greatly congruent with the reference tree that coincides with the taxonomy accepted by biologists for these Proteobacteria.

**Conclusions:**

In this study, we have introduced a new web server OGtree2 at http://bioalgorithm.life.nctu.edu.tw/OGtree2.0/ that can serve as a useful tool for reconstructing more precise and robust genome trees of prokaryotes according to their overlapping genes.

## Background

The approach to analyzing a single gene (e.g., ribosomal RNA) has proved itself to be a powerful tool in molecular phylogenetic studies. However, it may not be suitable for deriving the phylogenetic history of organisms because it sometimes provides insufficient resolution in the derived tree due to limited phylogenetic information in a single gene, or it gives rise to conflicting trees when applied to different individual genes due to different evolution rates or horizontal gene transfer [1]. The recently advent of high-throughput sequencing techniques has made it possible and reliable for evolutionary biologists to reconstruct phylogenetic trees of organisms (hereafter called *genome trees*) using the overwhelming amount of genomic information extracted from their complete genomes. It is also believed that the created genome trees are less affected by variable mutation rates or horizontal gene transfer events. So far, many different methods on the basis of this principle have been proposed [2], such as gene content based on the presence and absence of genes [3,4] and gene order based on the presence and absence of gene pairs [5-7]. Gene order basically evolves faster than gene content [2]. To gain a high-resolution genome tree, therefore, gene order is more suited for closely related organisms, whereas gene content is more suited for distantly related organisms.

Recently, Luo *et al*. [8,9] have proposed a new method, as well as a server named BPhyOG, for reconstructing the genome trees of some prokaryotes only based on the content of overlapping genes. The so-called *overlapping genes *(OGs) are defined as adjacent genes whose coding sequences (CDSs) overlap partially or entirely. In their studies [8,9], Luo *et al*. have reported that OGs can serve as a useful phylogenetic character by providing interesting additional insights into phylogenetic relationship among prokaryotes. Their rationale for doing this is as follows. As phylogenetic characters, OGs may not evolve as slowly as gene content, because they can be observed frequently in all prokaryotic genomes and may also mutate at a universal (constant) rate [10,11]. On the other hand, OGs have more evolutionary conservation than gene order because the linkage may be preserved between two functional-related OGs [12-14]. However, we have found that some prokaryotic genome trees constructed using BPhyOG are not greatly consistent with those produced by traditional phylogenetic approaches based on ribosomal RNAs and/or concatenation of multiple protein sequences [15].

To address this problem, we have recently implemented a new server, called OGtree [15], which allows evolutionary biologists to reconstruct more reliable genome trees of some prokaryotes by using not only their OG content but also their OG order. It has been widely accepted that during evolutionary course, species genomes are subject to rearrangements, such as reversals (also called inversions), transpositions and translocations, all of which can alter the order and/or the orientation of genes in the genomes. As a consequence, the orders of orthologous OG pairs even between two closely related organisms may not be conserved. This suggests that we should take into account both OG content and orthologous OG order when reconstructing the genome trees of prokaryotes using the information of OGs. In our previous study [15], therefore, we have defined an *overlapping-gene distance *between two genomes based on a measure of combining OG content (i.e., the presence and absence of OGs) and OG order (i.e., the presence and absence of orthologous OG pairs) in their whole genomes and also implemented our OGtree according to the pairwise OG distances between prokaryotic genomes. Our experimental results for a set of closely related Proteobacteria showed that our OGtree outperformed BPhyOG in the quality of reconstruction of their genome trees.

In this study, we further improve the accuracy of our OGtree by extending the genes retrieved from their complete genomes to include their regulatory regions and redefining the distance measure between two orthologous OG orders using genome rearrangements rather than breakpoints caused by the absence of orthologous OG pairs. The reasons for doing so are as follows. For some distantly related prokaryotic genomes, the amount of their overlapping CDSs is limited so that it is hard to find enough OG pairs to properly evaluate their pairwise OG distances and accurately reconstruct their genome trees. Actually, the term "gene" defined in modern genomics should include not only its coding region, but also its regulatory regions, such as promoter (at the 5' upstream end of the coding region) and terminator (at the 3' downstream end of the coding region) [16]. In addition, overlapping of regulatory regions of distinct genes should be of certain interest, because the regulation of expression for these genes is more or less interrelated [17]. In this study, therefore, we expand the region of a gene to include both its CDS and regulatory regions so that two adjacent genes whose CDSs or regulatory regions overlap with each other are considered as a pair of overlapping genes.

On the other hand, the orders of orthologous OG pairs between two prokaryotic genomes, as mentioned above, are often different due to genome rearrangements. The distance measure between two orthologous OG orders we previously defined was analogous to the breakpoint distance between two gene orders, which has been widely used as a rough measure of genomic distance [5]. In contrast to the genome rearrangement distance, however, the breakpoint distance may not correspond to an optimal series of events that accounts for the rearrangements of one genome with respect to another. Moreover, it is still not clear how to adapt the breakpoint analysis to multi-chromosomal genomes [18]. In this study, therefore, we try to use the genome rearrangement distance involved with reversals, block-interchanges (i.e., generalized transpositions) and translocations [19,20] to re-define the distance of the orthologous OG orders between two prokaryotic genomes.

## Results and Discussion

**Figure 1:**
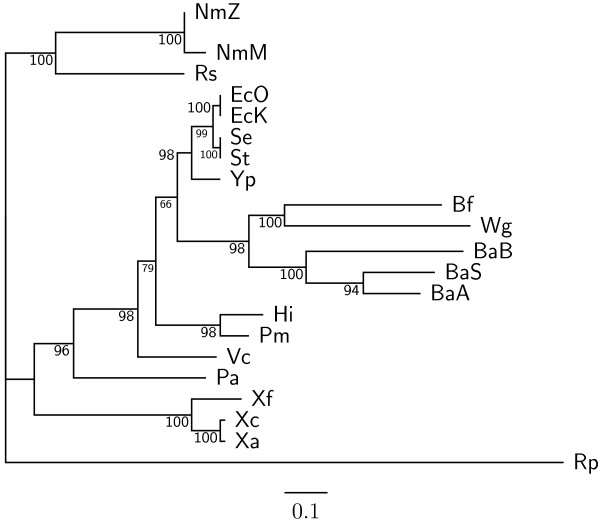
**Phylogenetic tree obtained from a trimmed alignment of 60 concatenated homologous proteins using maximum likelihood method with support values on its branches, which was adapted from **[21].

**Figure 2:**
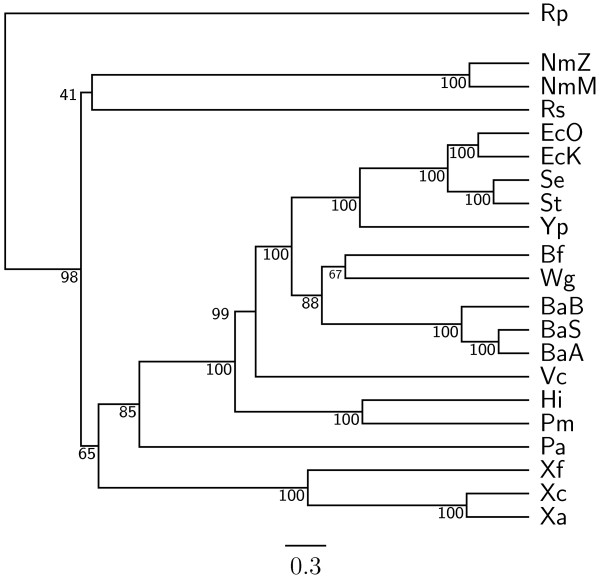
**Genome tree obtained using OGtree2 with UPGMA method**. The numbers on the branches are jackknife support values from 1,000 replicates.

**Figure 3:**
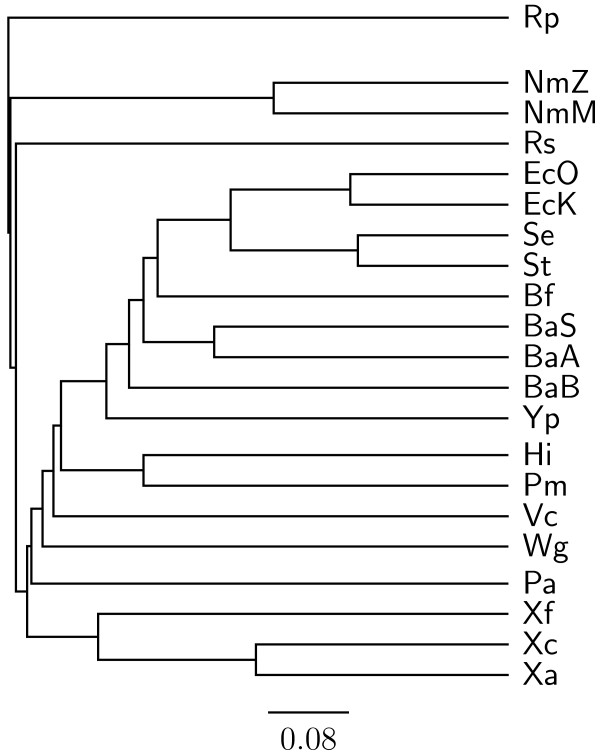
**Phylogenetic tree constructed using BPhyOG **[8,9].

**Figure 4:**
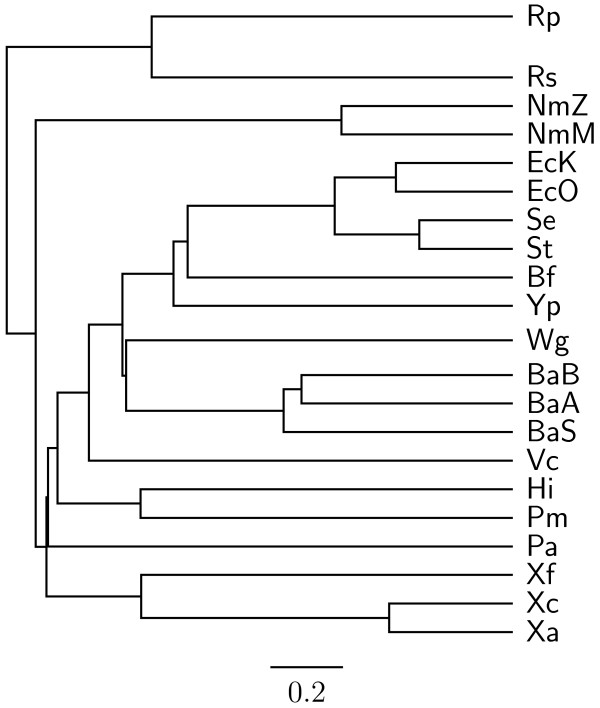
**Genome tree obtained using OGtree with UPGMA method **[15].

**Figure 5:**
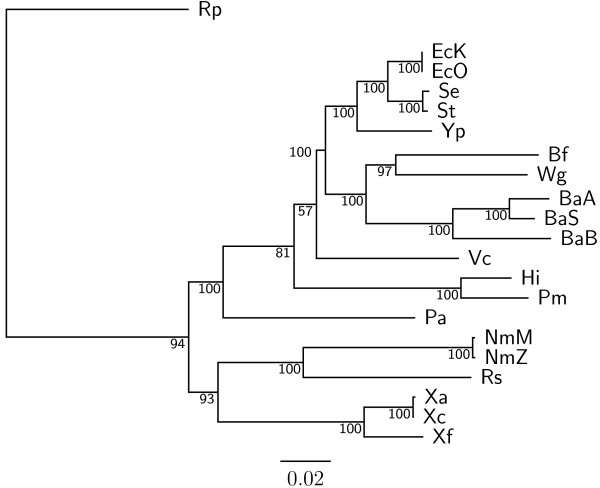
**Phylogenetic tree obtained from 16s rRNAs using the neighbor joining method**. The numbers on the branches are bootstrap support values from 1,000 replicates.

To demonstrate the accuracy improvement achieved by our new OGtree2, we have selected 21 genomes of Proteobacteria, which consist of one *α*-Proteobacteria, three *β*-Proteobacteria and 17 *γ*-Proteobacteria, as the testing dataset (Table 1). This dataset was previously used by Comas *et al*. [21] for their phylogenomic study on the monophyletic origin of insect endosymbionts from the *γ*-Proteobacteria, a debated issue with several conflicting reports. In addition, we used the phylogenetic tree constructed by Comas *et al*. [21] based on concatenated sequences of 60 homologous proteins as a reference tree (Figure 1) and compared the genome tree obtained by our OGtree2 (Figure 2) to those phylogenetic trees predicted by BPhyOG (Figure 3) [8] and our previous OGtree (Figure 4) [15]. As was argued in [21], the phylogenetic tree in Figure 1 can be considered as a good reference tree because it coincides with the taxonomy accepted by biologists for these Proteobacteria. In particular, the three *Buchnera *species in this reference tree form a monophyletic group with the other insect endosymbionts of *B. floridanus *and *W. glossinidia*. In addition, this group of endosymbionts is a sister clade to the cluster of the other five enterobacteria of *Yersinia*, *Esherichia *and *Salmonella*. However, it is worth mentioning here that the phylogenetic tree created by using 16S rRNAs, as shown in Figure 5, is different from that in Figure 1. In this 16S rRNA tree, the *γ*-Proteobacteria of *X. axonopodis*, *X. campestris *and *X. fastidiosa *were placed in the *β*-Proteobacteria branch and the species of *V. cholerae *was placed away from *P. aeruginosa*. This evidences that the single-gene approach to analyzing the 16S rRNAs is not suitable for inferring the phylogenetic relationships between these Proteobacterial organisms.

**Table 1:** Complete genomes of 21 Proteobacteria used in this study.

**Abbrev**.	Species (strain)	**Accession no**.	Division	Order
Rp	*Rickettsia prowazekii*	NC_000963	*a*	Rickettsiales
Rs	*Ralstonia solanacearum*	NC_003295	*β*	Bulkholderiales
NmM	*Neisseria meningitidis *MC58	NC_003112	*β*	Neisseriales
NmZ	*Neisseria meningitidis *Z2491	NC_003116	*β*	Neisseriales
EcK	*Escherichia coli *K12	NC_000913	*γ*	Enterobacteriales
EcO	*Escherichia coli *O157:H7 EDL933	NC_002655	*γ*	Enterobacteriales
Se	*Salmonella enterica *subsp. *enterica *serovar Typhi Ty2	NC_003198	*γ*	Enterobacteriales
St	*Salmonella typhimurium *LT2	NC_003197	*γ*	Enterobacteriales
Yp	*Yersinia pestis *KIM	NC_004088	*γ*	Enterobacteriales
Bf	*Blochmannia floridanus*	NC_005061	*γ*	Enterobacteriales
BaB	*Buchnera aphidicola *str. Bp	NC_004545	*γ*	Enterobacteriales
BaS	*Buchnera aphidicola *str. Sg	NC_004061	*γ*	Enterobacteriales
BaA	*Buchnera aphidicola *str. APS	NC_002528	*γ*	Enterobacteriales
Wg	*Wigglesworthia glossinidia brevipalpis*	NC_004344	*γ*	Enterobacteriales
Vc	*Vibrio cholerae *El Tor N16961 (I)	NC_002505	*γ*	Vibrionales
	*Vibrio cholerae *El Tor N16961 (II)	NC_002506	*γ*	Vibrionales
Hi	*Haemophilus influenzae *Rd	NC_000907	*γ*	Pasteurellales
Pm	*Pasteurella multocida *Pm70	NC_002663	*γ*	Pasteurellales
Pa	*Pseudomonas aeruginosa*	NC_002516	*γ*	Pseudomonadales
Xa	*Xanthomonas axonopodis *pv. *citri *str. 306	NC_003919	*γ*	Xanthomonadales
Xc	*Xanthomonas campestris *pv. *campestris *str. ATCC 33913	NC_003902	*γ*	Xanthomonadales
Xf	*Xylella fastidiosa*	NC_002488	*γ*	Xanthomonadales

**Figure 6:**
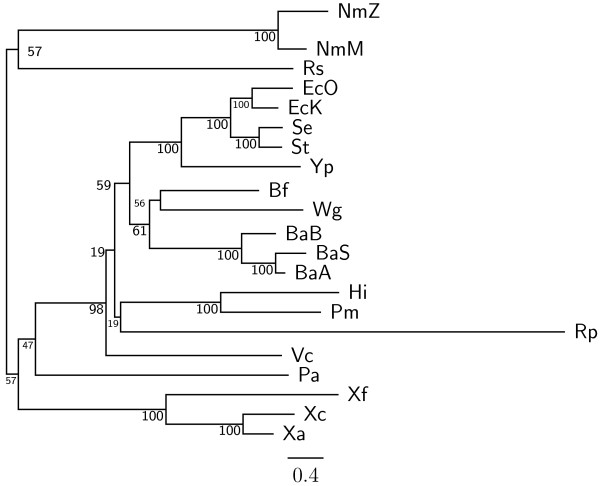
**Genome tree obtained using OGtree2 with NJ method**. The numbers on the branches are jackknife support values from 1,000 replicates.

**Figure 7:**
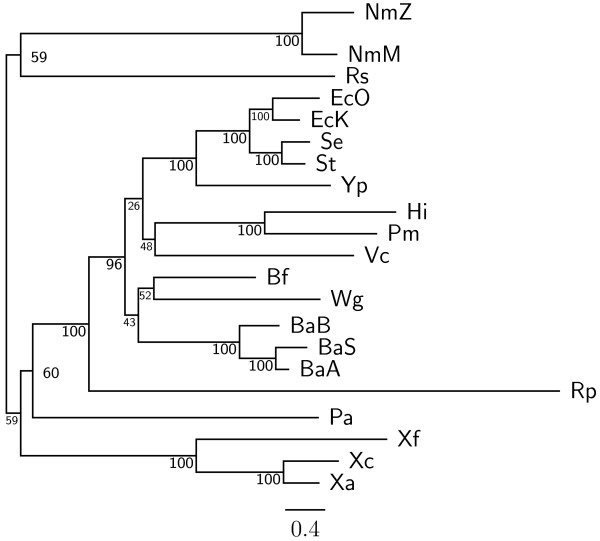
**Genome tree obtained using OGtree2 with FM method**. The numbers on the branches are jackknife support values from 1,000 replicates.

In our experiment, the topologies of both the neighbor-joining (NJ) and Fitch-Margoliash (FM) trees (see Figures 6 and 7, respectively) we obtained using OGtree2 were different from the one in the unweighted pair group method with arithmetic mean (UPGMA) tree (see Figure 2) with respect to the positions of *R. prowazekii*, *V. cholerae*, *H. influenzae *and *P. multocida*. Particularly, the *α*-Proteobacterium *R. prowazekii *was placed in the branch of *γ*-Proteobacteria in both the NJ and FM trees. The two Pasteurellaceae species (i.e, *H. influenzae *and *P. multocida*) and *V. cholerae *were neighbors in the NJ tree, while in the FM tree they formed a monophyletic group that was placed in the enterobacterial branch. As to the UPGMA tree, its topology was greatly congruent with that of the reference tree as shown in Figure 1. In particular, the UPGMA tree clearly and correctly divided the 21 Proteobacteria into three monophyletic clades and it also reflected monophyly not only for the three *Buchnera *species but also for a wider group including the other insect endosymbionts of *B. floridanus *and *W. glossinidia *with higher support values. However, *V. cholerae *in the UPGMA tree was placed a little away from *P. aeruginosa*, which is the same as the phylogenetic tree of 16S rRNAs in Figure 5. As done in our previous study on OGtree [15], as well as the studies by Luo *et al*. on BPhyOG [8,9], the UPGMA method in this experiment produced a genome tree that is much more congruent with the reference tree constructed using a trimmed alignment of 60 concatenated protein sequences, when compared to both the NJ and FM methods. This characteristic may be due to that, as originally reported in [10,11], evolution of OGs occurs at a constant mutation rate across bacterial genomes, suggesting that the UPGMA method is more suitable than both the NJ and FM methods for the reconstruction of prokaryotic phylogenies on basis of OG pairs.

As compared to the phylogenetic tree constructed by BPhyOG (Figure 3), our OGtree2 produced a more accurate phylogeny (Figure 2) for the 21 Proteobacteria genomes used in this study. In the BPhyOG tree, the relationship of endosymbionts was paraphyletic, because the three *Buchnera *species failed to form a monophyletic group and the two insect endosymbionts, *W. brevipalpis *and *B. aphidicola*, were separated far away from each other. In addition, the three *β*-Proteobacteria were placed just as neighbor taxa rather than a sister cluster. In contrast, *W. brevipalpis*, *B. aphidicola *and other three *Buchnera *species in our UPGMA tree (Figure 2), as well as in the reference tree (Figure 1), were placed as a sister group, suggesting that there should be a common origin for these five endosymbionts. Moreover, our current OGtree2 indeed outperformed over its previous version OGtree in phylogeny reconstruction for prokaryotes, because in the genome tree predicted by OGtree using the UPGMA method (Figure 4), the *α*-Proteobacteria of *R. prowazekii *and the *β*-Proteobacteria of *R. solanacearum *were placed together as a sister group and the insect endosymbiont of *B. floridanus *was placed in the branch of enterobacteria.

As demonstrated above, as well as in other previous studies [8,9,15], OG pairs indeed can serve as a useful tool in phylogenetic inference of prokaryotes, because they are abundant and more conserved than non-overlapping genes in prokaryotic genomes and even may evolve at a constant rate across prokaryotic genomes. In fact, our algorithm for constructing the genome trees of prokaryotes using OG pairs relies on successfully identifying orthologous genes, as well as authentic ORFs and horizontally transferred genes, before we can compare the OG content and order across organisms and calculate their pairwise OG distances. Therefore, more accurate identification of authentic ORFs, HGT events and orthologous genes will definitely further improve the accuracy of our algorithm and software tool. On the other hand, we measured the OG distance by taking into account of both the OG content and order in a pair of organisms. Particularly, we estimated the OG order distance (i.e., *r*_*i,j*_) by using the genome rearrangements involved with reversals (or inversions), block-interchanges (i.e., generalized transpositions) and translocations (including fusions and fissions) [19,20]. Although this rearrangement distance may underestimate the true distance, we believe that the difference between them for the Proteobacteria we used in this study is small. The reasons for this small difference are as follows. First, the rearrangements we considered include not only reversals but also transpositions and translocations. Second, Bourque and Pevzner [18] have conducted simulations to compare the estimated reversal distances and the true ones, consequently showing that the reversal distance approximates the true distance very well as long as the number of reversals remains below 0.4 *n *(i.e., the normalized reversal distance is less than or equal to 0.4), where *n *is the number of genes being considered. According to the experimental results we obtained in this study, the normalized rearrangement distance (i.e., *r*_*i,j*_/*n*) typically varies from 0 to 0.12 for closely related prokaryotes (e.g., free-living Enterobacteriaceae, while it is typically in the range of 0.43 to 0.5 for more divergent organisms (e.g., between Pasteurellaceae and free-living Enterobacteriaceae).

## Conclusions

Previously, we have implemented a web server named OGtree to demonstrate that overlapping genes can be served as a useful genomic marker for reconstructing genome trees of some prokaryotes. In contrast to BPhyOG, the OG distance we defined to measure the difference between two prokaryotic genomes in our OGtree was based on a combination of their OG content and orthologous OG order. In this study, we have further improved the accuracy of our OGtree in reconstruction of prokaryotic genome trees by extending the regions of genes to include their regulatory regions and redefining the distance measure between two orthologous OG orders using genome rearrangements rather than simple breakpoints. Our experimental results on a set of 21 Proteobacteria have also shown that the above modifications indeed helped us to reconstruct a more precise and robust genome tree that coincides with the taxonomy accepted by biologists for these Proteobacteria. This suggests that our current OGtree2 can provide interesting insights into the study of evolutionary relationships of completely sequenced prokaryotic genomes.

## Methods

### Algorithm of OGtree2

Basically, OG pairs are classified into three directional patterns, namely, *unidirectional *(→→), *convergent *(→←), and *divergent *(←→). It was reported that in prokaryotic genomes unidirectional OGs are most abundant, convergent OGs are less common, and divergent OGs are rare [10,11,22]. We define the so-called *orthologous OG pairs *from two different genomes *G*_*i *_and *G*_*j *_as pairs of genes that overlap in *G*_*i *_and have orthologous counterparts with the same directional pattern that also overlap in *G*_*j*_. Suppose that there are totally *n *orthologous OG pairs between *G*_*i *_and *G*_*j*_. Then we define the *overlapping-gene distance D*_*i,j *_between *G*_*i *_and *G*_*j *_as follows.

In the above formula, *r*_*i,j *_denotes the genome rearrangement distance between *G*_*i *_and *G*_*j *_using reversals, block-interchanges (i.e., generalized transpositions) and translocations (including fusions and fissions), which can be computed in polynomial time when block-interchanges are weighted 2 and the others are weighted 1 [19,20], and *x*_*i *_and *x*_*j *_denote the numbers of total OGs in *G*_*i *_and *G*_*j*_, respectively. Basically, *D*_*i,j *_evaluates the distance between *G*_*i *_and *G*_*j *_by considering the orthologous OG order measure as defined in the first term and the OG content measure as defined in the second term. Then *w*_*o *_and *w*_*c *_can be considered as the weight of orthologous OG order and the weight of OG content, respectively, where their defaults are now set to 1 and 2, respectively, in our OGtree2.

In theory, it can be proved that the value of *r*_*i,j *_is less than or equal to *n *[20]. Below, we give an intuitive reason for this property. Basically, the number of breakpoints between two permutation orders over the same set of *n *OG pairs is less than or equal to *n*. In the generic case, an optimal reversal or translocation will remove two breakpoints, while an optimal block-interchange will remove four breakpoints. We therefore have *r*_*i,j *_≤ *n*, even though reversals and translocations are weighted 1 and block-interchanges are weighted 2. It is also worth mentioning, according to the experimental results we obtained in this study, that for closely related prokaryotes (e.g., free-living Enterobacteriaceae), *r*_*i,j *_is typically between 0 and 120, while for more divergent organisms (e.g., between Pasteurellaceae and free-living Enterobacteriaceae), *r*_*i,j *_is typically in the range of 170 to 217, and typical values for *n *range between 13 and 2,700 depending on how closely related the species are.

**Figure 8:**
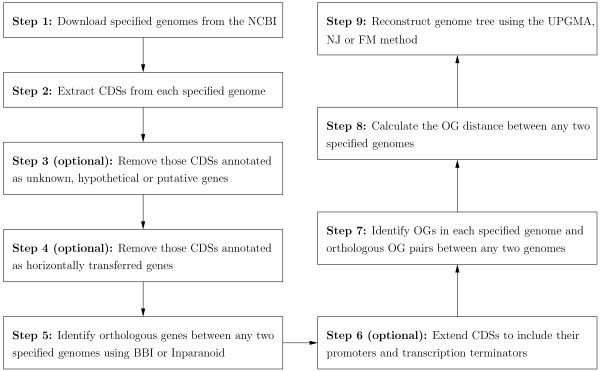
**Basic pipeline of reconstructing genome trees using OG pairs**.

In the following, we describe the details about the procedures, as shown in Figure 8, we used to develop our new web server, named OGtree2, for reconstructing genome trees of prokaryotes using their OG pairs. First, we download complete genomes from the National Centre for Biotechnology Information (NCBI) using the accession numbers specified by the user. The putative genes are then extracted from each of these downloaded genomes based on the annotation of coding sequences (CDSs). Inevitably, some of these putative genes may not be annotated correctly and, therefore, we allow the user to further exclude those annotated as unknown, hypothetical or putative genes for a more reliable analysis. In addition, we offer an option in our OGtree2 to remove those annotated as horizontally transferred genes at the HGT-DB database [23], because they are very common in prokaryotic genomes and may obscure the OG pairs with which we hope to reconstruct the genome tree of prokaryotes.

Next, the BLASTP program [24] is used to determine putative orthologous genes between two genomes according to the so-called bidirectional best hit (BBH) approach. A BBH denotes a pair of genes *a *and *b *from two genomes *G*_*i *_and *G*_*j *_such that *b *is the best hit (i.e., most similar gene) when *a *is compared against all genes of *G*_*j*_, and vice versa. Tatusov *et al*. [25] have previously evidenced that the BBH approach works reasonably well for identifying putative orthologs of bacterial genomes. The Inparanoid program [26] is also used as an alternative to identify putative orthologous genes between any two genomes, because it has been shown to be the best among five currently existing methods of automatically detecting orthologous genes [27]. Recall that the term "gene" defined in this study can be expanded to include not only its coding region but also regulatory regions, such as promoters and transcription terminators. Basically, the promoters of prokaryotes are always located immediately upstream of the transcription start site (TSS), the TSSs are located upstream of the start codon, and the transcription terminators are located downstream of the stop codon. In this case, the CDSs of genes are further extended at their 5' and 3' ends to their regulatory promoter and terminator regions. Then two adjacent genes in each genome are identified as overlapping genes (OGs), or an OG pair, if their CDSs (or extended CDSs) overlap partially or completely. Two OGs, say (*a, c*) and (*b, d*), from different genomes are then considered as an orthologous OG pair if *a *and *b*, as well as *c *and *d*, are orthologous to each other, and (*a, c*) and (*b, d*) have the same directional pattern.

Finally, we calculate the pairwise OG distance *D*_*i,j *_between any two given genomes *G*_*i *_and *G*_*j *_according to their OG pairs and then construct genome trees of all input genomes using the so-called distance-based methods of building trees, such as unweighted pair group method with arithmetic mean (UPGMA), neighbor-joining (NJ) and Fitch-Margoliash (FM).

Based on the algorithm described above, we have implemented a web server named as OGtree2 (http://bioalgorithm.life.nctu.edu.tw/OGtree2.0/) that allows the user to reconstruct prokaryotic genome trees with overlapping genes retrieved from the prokaryotic genomes.

### Orthologous OG Pair Identification and Genome Tree Reconstruction

It is inevitable that some genes may be misannotated in the genomes downloaded from the NCBI. We may therefore remove those CDSs annotated as unknown, hypothetical or putative genes from each downloaded genome in our analysis. As was done in [15], however, the fact that most of the CDSs in *W. brevipalpisa *are currently annotated as unknown, hypothetical or putative leads us to find no orthologous OG pair between *W. brevipalpisa *and other Proteobacteria, if all these CDSs in *W. brevipalpisa *are excluded from our analysis in this study. Instead, we first removed those horizontally transferred genes currently annotated at the HGT-DB database [23] and then used the BBH approach, as mentioned previously, to identify putative orthologous genes by setting the parameters with a minimum E-value of 10^-8^, at least 85% of each authentic CDS sequence involved in the alignment, and a minimum similarity of 45%. In addition, we observed that the amount of the orthologous OG pairs between non-*γ*-Proteobacteria genomes and other Proteobacteria genomes is few, resulting in difficulty in measuring the accurate OG distances between them. Recall that the term "gene" can be expanded to include both of its coding and regulatory regions, such as promoters and transcription terminators. In prokaryotic genomes, a promoter region, which basically contains the so-called *-*10 hexamer, extended *-*10 element, *-*35 hexamer and UP element, usually occupies about 60 base pairs (bp) upstream of the transcription start site (TSS) [28,29] and a terminator region usually occupies about 50 bp downstream of the stop codon [30]. Moreover, as exemplified in *E. coli *genome, 95% of TSSs occur 325 bp upstream from the translation start sites (TLS) of their corresponding genes [31]. According to these information, therefore, we extended the region of each CDS by 385 bp at its 5' end and by 50 bp at its 3' end, so that any two adjacent genes in a genome were considered as an OG pair if their extended CDSs partially or completely overlap with each other. With default values for all the other parameters (i.e., *w*_*c *_= 2 and *w*_*o *_= 1), we used OGtree2 to calculate the OG distance between every pair of Proteobacteria and finally construct the genome trees for all the Proteobacteria used in this study with the UPGMA, NJ and FM methods.

In prokaryotic genomes, many genes are organized into operon structures [32,33], where an *operon *is a cluster of genes co-transcribed in a single mRNA. Most operons typically have a single promoter located upstream of the first gene of operon and a single terminator located downstream of the last gene of operon. By the gene definition we used in this study, any two adjacent genes in such operons can be considered an OG pair because they have the same regulatory elements. In this situation, our method described above to identify OG pairs can still find most of them because it has been reported that, in most bacterial genomes, intergenic distances between genes in the same operon are often small (e.g., less than 20 bp) [10,32].

### Consensus Tree Reconstruction

To demonstrate the robustness of our method, we have adopted a method similar to the so-called jackknife resampling approach [34] to compute the support values of the tree branches as described as follows. We first randomly removed *e*^-1 ^≈ 37% of the initial OG pairs from each genome, while retaining the relative orders of the remaining OG pairs, and then calculated the OG distance between every pair of Proteobacteria. In this process, we implemented 1,000 such jackknife random samples to obtain 1,000 pairwise OG distance matrices. Next, we applied the NEIGHBOR/FITCH program in the PHYLIP package [35] to these 1,000 OG distance matrices to obtain 1,000 jackknife trees. Finally, we applied the CONSENSE program in the PHYLIP package to these 1,000 jackknife trees to obtain a majority-rule consensus tree with the numbers at each node representing the percentage of times that the clade defined by that node appears in the 1,000 jackknife trees.

### 16S rRNA Tree Reconstruction

We used the following procedure to obtain a 16S rRNA tree topology for the 21 Proteobacteria. First, the 16S rRNA sequences of these 21 Proteobacteria were downloaded from RDP (http://rdp.cme.msu.edu/)[36]. Their multiple sequence alignment were then obtained using CLUSTALW 1.8 [37]. Finally, the neighbor-joining tree was inferred using the NEIGHBOR program of PHYLIP 3.6 [35] and its support values were obtained by bootstrap resampling with 1,000 replicates.

## Authors' contributions

CLL conceived of the study, participated in the design and analysis of algorithm and drafted the manuscript. HTC participated in the analysis and interpretation of data and experimental results, as well as in drafting the manuscript. CHC and CHY participated in the software development and carried out the bioinformatics experiments. Basically, CHC and CHY contributed equally to this work and should be considered co-first authors. All authors read and approved the final manuscript.
